# Human Papilloma Virus Positive Oropharyngeal Squamous Cell Carcinoma and the Immune System: Pathogenesis, Immunotherapy and Future Perspectives

**DOI:** 10.3390/ijms25052798

**Published:** 2024-02-28

**Authors:** A. Khoo, M. Boyer, Z. Jafri, T. Makeham, T. Pham, L. M. Khachigian, P. Floros, E. Dowling, K. Fedder, D. Shonka, J. Garneau, C. H. O’Meara

**Affiliations:** 1 Department of Otolaryngology, Head & Neck Surgery, Canberra Health Services, Canberra, ACT 2601, Australia; 2 Chris O’Brien Lifehouse, Camperdown, NSW 2050, Australia; michael.boyer@lh.org.au; 3 Vascular Biology and Translational Research, Department of Pathology, School of Biomedical Sciences, Faculty of Medicine and Health, University of New South Wales, Sydney, NSW 2052, Australia; 4 ANU School of Medicine & Psychology, Australian National University, Canberra, ACT 0200, Australia; 5 St Vincent’s Hospital, 390 Victoria Street, Sydney, NSW 2010, Australia; 6 Department of Otolaryngology, Head & Neck Surgery, University of Virginia School of Medicine, Charlottesville, VA 22903, USA; urt4et@uvahealth.org (E.D.); klf2e@uvahealth.org (K.F.); jcg6j@uvahealth.org (J.G.)

**Keywords:** head & neck cancer, immunotherapy, immune system, cancer of the oropharynx

## Abstract

Oropharyngeal squamous cell carcinoma (OPSCC), a subset of head and neck squamous cell carcinoma (HNSCC), involves the palatine tonsils, soft palate, base of tongue, and uvula, with the ability to spread to adjacent subsites. Personalized treatment strategies for Human Papillomavirus-associated squamous cell carcinoma of the oropharynx (HPV^+^OPSCC) are yet to be established. In this article, we summarise our current understanding of the pathogenesis of HPV^+^OPSCC, the intrinsic role of the immune system, current ICI clinical trials, and the potential role of small molecule immunotherapy in HPV^+^OPSCC.

## 1. Introduction

Oropharyngeal squamous cell carcinoma (OPSCC) is a subset of head and neck squamous cell carcinoma (HNSCC) involving the palatine tonsils, soft palate, base of tongue, and uvula, with a common capacity to spread to adjacent subsites. For decades, it was assumed that all OPSCC cases were aetiologically homogenous. However, in 1983, HPV antigens were discovered in a subset of OPSCC tumours [1]. Subsequent investigation has improved our understanding of this disease process and defined HPV^+^OPSCC as a disease process distinct from HPV^−^OPSCC, with differing epidemiological, genetic, and prognostic traits [2].

By way of example, genetic analyses have identified mutations in PIK3CA and FGFR pathways in HPV^+^OPSCC and overexpression of CDKN2, encoding for p16 [3,4,5]. In contrast, HPV^−^OPSCC is characterised by mutation of tumour suppressor genes (i.e., TP53), EGFR upregulation, and low p16 expression [3,4,5]. Furthermore, at the cellular level, there is a distinct difference between tumour microenvironments (TME) and associated immune cell infiltrates [6]. The clinical presentation of HPV^+^OPSCC patients is also very different. Simplistically, HPV^+^OPSCC patients are commonly younger, non-smokers/drinkers, presenting with enlarged non-tender cervical lymphadenopathy and an unknown primary (low T-stage, high N-stage tumours) [7,8,9,10,11], whilst HPV^−^OPSCC patients are older, smokers/drinkers, often presenting with a larger painful primary tumour causing dysphagia or odynophagia and late-stage cervical lymphadenopathy (high T-stage, low N-stage tumours) [12,13,14,15]. Importantly, this disparity has recently helped to drive clinical and radiomic diagnostic techniques [16,17]. Additionally, HPV^+^OPSCC is more sensitive to current standard of care therapies (specifically non-surgical regimes) and has a superior prognosis over HPV^−^OPSCC for any stage [18,19]. Consequently, the 8th edition of the AJCC TNM staging system acknowledged this and defined HPV^+^OPSCC as a distinct clinical entity [20].

Despite this, personalized treatment strategies specific for HPV^+^OPSCC are yet to be established. Currently, the standard of care intervention for OPSCC involves surgery, radiotherapy, or chemotherapy as either uni- or multi-modality therapy that is stage-dependent. As this is applied irrespective of HPV status, it does not account for the improved prognosis typically seen with HPV^+^OPSCC patients. This exposes patients to increased survivorship morbidity and a 15–40% risk of radiation-induced metachronous primary in a younger demographic [21]. Radiotherapy de-escalation trials are currently recruiting to ascertain the ability of dose reduction in HPV^+^OPSCC, which is exquisitely radiosensitive, to maintain high rates of cure, preserve quality of life, and reduce metachronous primary rates. Early trials are promising; however, study comparison has been difficult due to varied trial designs, further complicated by small samples sizes, treatment variability, non-uniform inclusion criteria, and long-term follow-up issues [22]. Despite this, these studies universally demonstrate that de-escalation is feasible and safe, helping to personalise the therapy of HPV^+^OPSCC [23]. It is important to note, however, that there is currently a recommendation away from dose de-escalation in heavy smokers and patients with increased alcohol consumption [24,25].

In a further step towards personalised and targeted treatment strategies, development of immune checkpoint inhibitors (ICI) has heralded a paradigm shift in HNSCC therapy. Targeting molecular “brakes” applied to the immune system, the release of these brakes can enable immunorecognition and promote immune-mediated tumour cytotoxicity, affording a small population of patients suffering recurrent or metastatic HNSCC (R/M HNSCC) a partial to complete response [26]. Trials are currently being completed to determine the efficacy of immunotherapy in HPV^+^OPSCC over standard-of-care regimes. This article explores our current insight into the pathogenesis of HPV^+^OPSCC, the intrinsic role of the immune system, current ICI clinical trials, and the potential role of small molecule immunotherapy to personalise treatment strategies further in HPV^+^OPSCC.

### 1.1. Epidemiology

The global incidence of OPSCC was over 3.7 million cases in 2020, 33% attributable to HPV [27,28], with the incidence of OPSCC rapidly increasing in developed countries when compared to all cancers [29,30]. This increase has been identified in the US, Europe, UK, Australia, New Zealand, and certain countries within Asia [28,31,32,33,34,35,36,37], with further studies confirming a rising trend across all countries [34,36,37,38,39,40,41,42]. HPV is the most common sexually transmitted infection (STI) and one of the most common viral infections in the world [43], and HPV^+^OPSCC has now achieved the notorious distinction of becoming the predominant HPV-related malignancy in the US and UK, superseding cervical cancer [44].

Early studies identified that HPV^+^OPSCC disproportionately affected males and a younger population when compared to HPV^−^OPSCC [30]. However, in a result likely to represent a ‘birth cohort effect’, recent studies have demonstrated a rise in the diagnostic age of HPV^+^OPSCC patients, with a significant increase over the age of 65 [30,45,46,47,48]. Specifically, one study found that the median age of diagnosis increased from 53 years in 1995–2000 to 58 years in 2001–2013 [49]. This demographic redistribution has important clinical implications for therapeutic delivery within the aging population.

### 1.2. Risk Factors

Risk factors for HNSCC have classically been identified to include tobacco smoking [50], alcohol consumption [51], betel quid chewing [52], and EBV infection [53]. Specific to the oropharyngeal subsite, oral infection with the high-risk HPV serotypes (HR-HPV), particularly serotypes 16 and 18, is highly correlated with OPSCC [54]. In comparison to HPV^−^OPSCC, HPV^+^OPSCC is not commonly associated with a history of tobacco smoking or alcohol use [18,55]. However, HPV^+^OPSCC in the setting of either heavy smoking or alcohol consumption is associated with a worse prognosis [25,56] that may be dose dependent in the setting of smoking [57]. Certainly, studies have identified that tobacco chemicals enhance viral oncogenic expression in HPV-infected cervical epithelium [58]. However, the relationship between these three risk factors (HPV, tobacco smoke, and alcohol) is poorly understood, with some studies demonstrating synergistic effects [59,60] while others have not identified this phenomenon [61,62].

While there was initially some evidence to suggest an association between high-risk sexual activity and development of HPV^+^OPSCC [54], these are not reproducible. Subsequent studies have found that neither the number of sexual partners nor oral sex practices are associated with OPSCC development when adjusted for potential confounders including age, gender, smoking, and alcohol consumption [63,64,65,66]. In contrast, in a finding universal to SCC, primary (i.e., epidermodysplasia verruciformis, WHIM Syndrome, DOCK8 mutations, GATA Binding protein mutations, and severe combined immunodeficiency, defects in NK-cells and lymphopenias) [67,68,69], acquired, or secondary immunodeficiencies (transplant recipients, iatrogenic immunosuppressant, and autoimmunity) are risk factors for HPV-driven malignancies [69,70,71]. Specifically, research has found that HIV-positive patients have 1.6–3.2 times higher risk of developing HPV^+^OPSCC [72,73].

### 1.3. Pathogenesis/Oncogenesis

HPV are 50–60 nm in diameter, non-enveloped, icosahedral double-stranded DNA (dsDNA) viruses comprising 8000 base pairs bound to cellular histones and contained in a protein capsid of 72 pentameric capsomers [74]. HPVs are highly epitheliotropic, infecting cutaneous and mucosal epithelial cells via an unconventional clathrin-independent endocytic pathway [75] and are strongly associated with cervical, head and neck, anogenital, and oesophageal cancers. Of the more than 200 HPV serotypes, 14 are characterised as HR-HPVs due to their carcinogenic potential, including HPV 16, 18, 31, 33, 35, 39, 45, 51, 52, 56, 58, 59, 66, and 68 [76]. For OPSCC specifically, HPV 16 has been identified in the majority of HPV^+^OPSCC cases, while HPV 18 and other HR-HPV serotypes have been identified in HPV^+^OPSCC cases, but are much less common [77]. This is distinct from the multiple oncogenic HPV serotypes associated with cervical cancer, specifically 16, 18, 31, 33, 45, and 52 [78].

**Figure 1:**
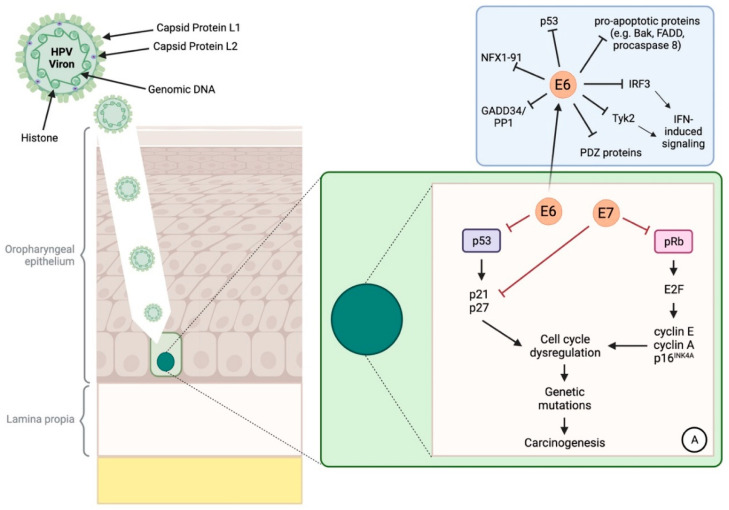
**HPV Patho-oncogenesis.** The HPV virion invades the epithelium of the oropharynx, infecting the basal layer of cells. Within these cells, E6 and E7 inhibit p53 (tumour protein 53) and pRb (retinoblastoma protein) respectively, affording the cell the ability to replicate without regulation by these Tumor Suppressor Genes (TSGs). Further to this E6, promotes carcinogenesis in several ways: firstly, through inhibition of pro-apoptotic proteins (namely, GADD34/PP1 (growth arrest and DNA damage induced transcript 34/serine/threonine protein phosphatase), Procaspase 8, FADD (FAS-associated death domain protein), or Bak) [85,86,87,88]; secondly, by suppressing host–IFN (interferon) antiviral response by downregulating IRF3 (interferon regulatory factor 3) and Tyk2 (Tyrosine kinase 2); thirdly, by disrupting tissue integrity through degradation of PDZ proteins (PDZ proteins play an important role in anchoring receptor proteins in the cell membrane to cytoskeletal components; these proteins also play an integral role in signal transduction complexes. Interaction with certain proteins promotes oncogenic potential) via expression of a PDZ protein binding motif (PSD-95/D1g/ZO-1) [89]; and finally, by promoting cellular immortalisation by targeting NFX1-91, which is an endogenously expressed transcriptional regulator of human telomerase reverse transcription (hTERT) (active in stem cells) that promotes telomerase induction and cellular immortalisation [90].

The viral genome of all HPV serotypes contains eight open reading frames (ORFs) that are transcribed from a single strand of DNA. The ORF is composed of three functional sections: the early € region encoding for E1, E2, E3, E4, E5, E6, and E7 genes required for viral replication, the late gene section encoding for structural proteins (L1 and L2) required for virion assembly, and a large non-coding section known as the long control region (LCR), containing *cis* elements crucial for transcription and replication of viral DNA [79]. Extensive research has implicated the E5, E6, and E7 genes as primary oncoproteins in HPV-associated carcinogenesis and tumour progression [74]. These oncoproteins each play a unique and integral role in supporting cellular proliferation and inhibiting apoptosis (refer Figure 1: HPV Patho-oncogenesis). Specifically, E5 downregulates antiviral responses via inhibition of cGAS-STING pathway, immunoproteasome function, and antigen processing [80]. On the other hand, E6 and E7 predominantly promote the degradation of p53 and pRb, respectively [16], both representing tumour suppressor genes (TSG) critical for maintaining adequate regulation of the cell cycle in the context of DNA damage [81]. pRb dysfunction reprograms the host cell to become more dependent on HPV oncogenes and p16INK4A, which in this context helps to subvert oncogene-induced cellular senescence [82]. Although based upon cervical cancer, a recent study has shown that tumour progression is positively correlated with viral load and HPV E7 oncoprotein expression [83]. HPV has also been found to induce epigenetic alteration, such as DNA hypomethylation and tumour suppressor gene hypermethylation, which may contribute to tumorigenesis [84]. In summary, these mechanisms allow HPV-infected cells to accumulate the hallmark properties of transformation and facilitate carcinogenesis.

### 1.4. Diagnostic Techniques

The gold standard diagnostic test for HPV^+^OPSCC is E6/E7 mRNA detection [91]; however, its labour-intensive nature and cost preclude it from widespread use. Instead, routine diagnosis makes use of p16 immunohistochemistry (IHC), utilising p16 protein expression as a surrogate marker for E7-mediated pRb degradation [92]. The 8th edition of the TNM staging system also relies on p16 expression to distinguish HPV^+^OPSCC [20]. While it is widely used for its high sensitivity and ease of use, p16 IHC has moderate specificity, which can produce false positives [93]. Hence, p16 IHC in combination with highly accurate HPV DNA PCR is recommended for optimised specificity [92].

### 1.5. Vaccination

Given the remarkable impact of HPV vaccines on cervical cancer rates in the last 17 years [94], the question has been raised as to whether this prophylactic intervention will confer similar protection for HPV^+^OPSCC. Certainly, recent studies have demonstrated that vaccinated patients are less likely to develop HPV associated oral infections, with resistance extending to serotype 16 [95,96]. Confirming the benefit of vaccination, a recent study indicated that unvaccinated patients have a 19 times greater risk of developing OPSCC [97].

Although gender neutral vaccination regimes are being implemented in many developed countries [98], there is a paucity of vaccination in developing countries, limited by resources and socioeconomic and cultural factors. In 2023, 140 countries registered implementation of HPV vaccination programs in females [99] and 43 with gender-neutral vaccination, all being developed countries except for Bhutan [100]. Globally, an estimated 17% of females and 5% of males aged 15 in 2022 had completed the full course of the vaccine, while 21% of females and 7% of males had received one dose [101]. In addition, even with the implementation of gender-neutral regimes, there remains a significantly lower proportion of males being vaccinated, 44% in developed and 5% in developing countries overall respectively [102]. Clearly, there is a significant gap between ideal and practical vaccination rates especially in the male population, who are more likely to develop OPSCC.

Additionally, due to the latency of OPSCC development at a median age of 58 years [49], it may take several decades for the recent changes to HPV vaccination to be reflected in the OPSCC incidence rates. In the United States, it is projected that HPV vaccination will have limited impact on OPSCC rates until 2045 given that the current vaccination programs do not encompass the older population, who remain at elevated risk [103]. This continues to be an evolving preventative strategy that should pay dividends in lowering OPSCC rates.

Unfortunately, prophylactic vaccines are ineffective against established HPV-infection. There is, therefore, a necessity for specifically targeted immunotherapy or therapeutic HPV vaccine for people who have already acquired HR-HPV [69]. It is also unclear whether prophylactic vaccination will provide ongoing efficacy in the immunocompromised population and further investigation in this area is needed.

## 2. Role of the Immune System

The immune system is a complex interwoven network of specialised cells and molecules, all working in synchrony as the body’s defence mechanism. Decades of cancer immunology research have identified inextricable links between the immune system and tumorigenesis, a ‘tug-of-war’ between ‘immunosurveillance’-guided tumour cell cytotoxicity versus tumour immune evasion mechanisms sustaining cellular escape and proliferation.

**Figure 2:**
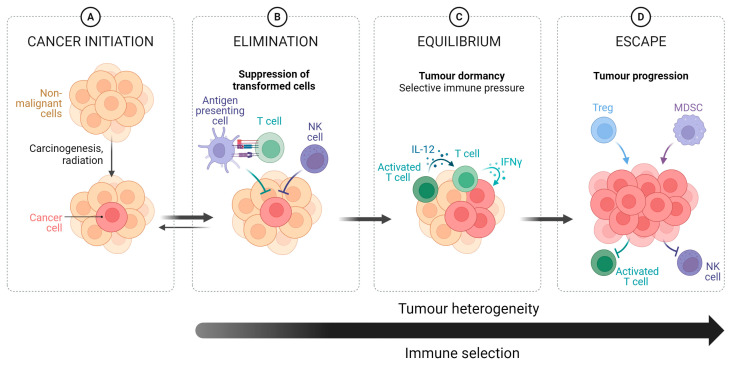
The different phases of the cancer immunoediting process. (**A**) Initiation of a carcinogenic process, producing cancer cells and development of a tumour. (**B**) Elimination is characterised by immunosurveillance leading to suppression of the transformed cancer cells directed by inflammatory and cellular cytotoxicity processes. (**C**) Equilibrium is characterised by a balance between the immune system and the tumour, leading to partial control of the tumour. This phase is characterised by high tumour mutational burden due to selective immune pressure. (**D**) Escape is caused by the increased selective pressure caused by the immune system leading the tumour to acquire immune evasion, resulting in uncontrolled tumour proliferation. Arrow indicates increasing Tumour heterogeneity and Immune selection. Figure adapted from “Cancer Immunoediting”, by BioRender.com (2023). Retrieved from https://app.biorender.com/biorender-templates, accessed on 10 February 2024.

Dunn and colleagues [104] developed a model, known as “immunoediting”, to help explain this phenomenon (refer to Figure 2 for a graphical overview). It postulates three phases of ‘cancer immune’ system interaction: *elimination*, *equilibrium*, and *escape*. *Elimination* represents successful interactions between innate and adaptive immune systems to eradicate tumour cells. A complex system, it relies upon direct recognition and cell-mediated cytotoxicity of tumour cells by NK-cells, tumour antigen uptake by antigen-presenting cells (dendritic cells and macrophages), and subsequent presentation and activation of T and B lymphocytes mediating inflammatory and cellular cytotoxicity processes to promote tumour cell cytotoxicity [105]. Antigen activation of the adaptive immune system has the added benefit of producing long-standing immunity. Activating the adaptive immune system promotes various downstream effects to support the innate immune system, destroy the tumour cells, and support systemic immunity and memory [106].

*Equilibrium* represents a balance between the immune system and the tumour. Only partial control of the tumour can be achieved, commonly caused by high mutational burden contributing to immune resistance. *Escape* is associated with uncontrolled proliferation of tumour cells, inadvertently supported by the immune system to acquire evasive properties. The ability to evade immune-mediated cytotoxicity was recognised as a hallmark of cancer in 2011 [107].

### 2.1. HPV & Immune System Evasion

Like other virally-associated malignancies, the immune system responds to HPV viral proteins. L1 capsid protein, E6, and E7 are primary antigens recognised by the immune system in HPV [108]. However, cervical cancer research has helped identify several immune evasion strategies that HPV employs in both the innate and adaptative immune system, helping to establish prolonged infection and increase the risk of tumorigenesis [109,110].

**Figure 3:**
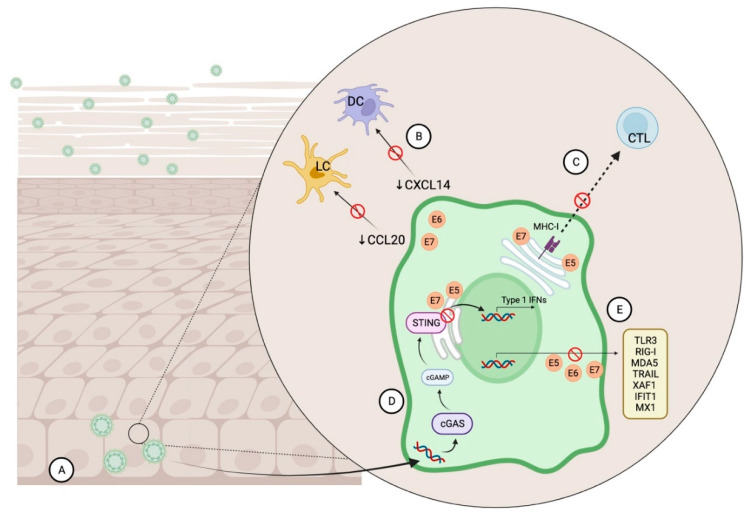
HPV’s immune evasion strategies. (**A**) HPV infects basal epithelial cells and utilises the progressive differentiation of keratinocytes to conceal its viral replication process from the immune system. Immune detection is prevented since the most viral gene expression happens in superficial epithelial layers and since no unexpected cell death or inflammation occurs. (**B**) HPV E6 and E7 oncogenes reduce secretion of chemokines, CCL20 and CXCL14, which hinders Langerhan’s and dendritic cell recruitment. (**C**) HPV E5 and E7 oncogenes prevent antigen presentation to cytotoxic T cells by downregulating major histocompatibility complex (MHC) class I expression. (**D**) HPV E5 and E7 oncogenes inhibit the cGAS-STING (cyclic GMP–AMP synthase, stimulator of interferon genes) pathway, which responds to aberrant DNA from viruses by activating the innate immune system. (**E**) HPV E5, E6, and E7 oncogenes can also inhibit IFN-stimulated gene expression (e.g., TLR3, RIG-I, MDA5, TRAIL, XAF1, IFIT1, MX1). cGAMP = 2′3′ cyclic GMP–AMP, CTL = cytotoxic T cell, DC = dendritic cell, IFN = Interferon, LC = Langerhan’s cell, MHC-I = major histocompatibility complex-I.

The first such mechanism involves minimising exposure to the immune system, starting from strategic infection of the basal layer of epithelium external to the basement membrane [108] (refer Figure 3: HPV’s Immune evasion strategies). HPV hijacks differentiating keratinocytes early in their evolution and completes its own viral replication cycle alongside this process. This means that the highest levels of viral gene expression and replication occur at the superficial epithelial layers, where immune cells are sparse [111]. Its virions are eventually shed alongside fully-differentiated keratinocytes during natural desquamation. This mechanism allows for HPV to infect host cells for a prolonged period without immune detection since it does not induce unexpected cell death, inflammation, or viraemia [110,112,113]. Because of this, poor anti-HPV humoral immunity and impaired T-cell immunity against HPV proteins are common in exposed individuals [114].

Secondly, HPV evades immune recognition by downregulating keratinocyte-induced alarm signals which indicate the presence of the pathogen to immune cells. One of these signals occurs via the cGAS-STING pathway, where cyclic GMP–AMP synthase (cGAS) detects viral dsDNA, activating stimulator of interferon genes (STING) protein [115]. STING recruits the innate immune system via the Type-1 interferon (IFN) antiviral response. Multiple HPV studies support HPV 16, 18 and E7 and E5 oncogene antagonism of STING [116,117], including within HPV+HNSCC cells [118,119], which helps the virus prevent immune activation. In addition, E6 and E7 oncogenes from HPV 16 have the ability to prevent keratinocyte secretion of NF-κB-dependent CCL20 [120,121] and CXCL14 [122] chemokines, interfering with Langerhan and dendritic cell migration respectively.

Thirdly, E5 and E7 can suppress antigen presentation and CD8+ cytotoxic T-lymphocyte activity via downregulation of major histocompatibility complex (MHC) class I expression [123]. Finally, HPV 16 E5, E6, and E7 oncogenes also have the capacity to inhibit IFN-stimulated gene expression for pathogen recognition receptors (TLR3, RIG-I, MDA5), for apoptosis (TRAIL, XAF1), for antiviral responses (IFIT1, MX1), and genes involved in the IFN pathway (STAT1) [124,125]. In combination, these evasive mechanisms enable HPV to persist in host cells, increasing the potential for malignant change.

### 2.2. HPV^+^OPSCC and the Tumour Microenvironment

Once HPV^+^ tumour cells develop, the immune system has been shown to play an integral role in supporting tumour progression and modulating the tumour response to therapeutic intervention. Innate and adaptive immune cells form a substantial part of the tumour microenvironment (TME), in addition to a diverse collection of non-malignant cells (including, but not limited to endothelial cells, fibroblasts, and adipose cells) forming the tumour stroma. Previously defined as passive, recent research has demonstrated the active impact of the TME in facilitating tumour growth and invasion [126,127].

HNSCC is one of the most highly immune cell-infiltrated tumours [128], a feature that has opened new avenues for personalised treatment using immunotherapy. The composition of the HNSCC immune infiltrate varies substantially between HPV^+^OPSCC and HPV^−^ OPSCC. HPV^+^OPSCC have greater densities of tumour-infiltrating lymphocytes (TIL), including CD3^+^ T cells, CD8^+^ T cells, Treg cells, B cells, and plasma cells, compared to HPV^−^OPSCC, but importantly and specifically B cells and CD8^+^ cytotoxic T-cells [129,130,131,132]. Additionally, HPV16 antigen-specific T cells with anti-tumour programs have been identified within immune infiltrates in 64% of HPV^+^OPSCC, providing a further potential personalised immunotherapy target in HPV^+^OPSCC [133].

The TME immune profile of HPV^+^OPSCC vs. HPV^−^PSCC has also been correlated with prognosis, which may explain improved overall survival (OS) and disease-free survival (DFS) in HPV^+^OPSCC [18,19,134,135]. High-density TIL infiltrate, especially involving CD4^+^, CD8^+^, and CD3^+^ subsets, has been associated with greater OS rates in HPV^+^OPSCC [136,137,138,139,140]. Higher levels of infiltrating CD20^+^ B cells also correlates with improved prognosis, specifically those expressing FCER2 which were localised to the stroma in HPV^+^HNSCC tumours [141,142]. FCER2^+^ B-cells have been shown to inhibit HPV^+^ tumour migration in vitro. FCER2 is an Fc receptor specific to IgE, upregulated on haemopoietic and B cells. Additionally, the pre-treatment neutrophil-to-lymphocyte (NLR) ratio in peripheral blood has been shown to inversely correlate with OS and DFS in HPV^+^OPSCC [143,144,145]. NLR is also an independent risk factor for severe disease [146] and neutrophilia is associated with increased expression of neutrophil extracellular traps (NETs). Increasing evidence indicates that NETs play a critical role within the TME (tumour associated neutrophils or “TANs” undergo polarisation to either N1 or N2 phenotypes in a similar fashion to macrophages, playing an important role in immunoediting [147]) and have been implicated in tumour progression, metastatic dissemination, and therapy resistance [148,149,150,151,152,153,154,155,156,157,158,159,160,161]. A recent genetic profile study investigating the clinical prognostic value of NET-related genes and their correlation to immunotherapy response identified that NETs gene expression can predict clinical outcomes and therapeutic response in HNSCC [162], whilst stromal NET density has been identified as an independent prognostic factor for recurrence-free survival (RFS) in cervical cancer, although an association with HPV-associated cervical cancer was not explored. This led to the suggestion that NET expression be added to TNM staging systems to help with prognostic stratification [163]. To date, there is a paucity of literature exploring the role of NETs in OPSCC, specifically HPV^+^OPSCC. Therefore, NET expression may represent a prognostic tumour biomarker and a target for development of immunotherapy agents.

Recently, the number of macrophages infiltrating into the TME have also been reported as an independent prognostic factor in HNSCC [164]. Tumour-associated macrophages (TAMs) can exacerbate desmoplasia, angiogenesis, nutrient deprivation, and immune suppression to promote tumour growth and regulate therapy resistance. Like neutrophils, the TME milieu can polarise macrophages to form M1 or M2 phenotypes (although this may ultimately prove to be an over-simplification). M1 phenotypes, in comparison to M2 cells, demonstrate an anti-tumour program, activating cytotoxic CD8+ T-cells and supporting the differentiation of CD4^+^ T-cells towards a Th-1 effector subset. Recent research has identified that HPV^+^OPSCC is associated with reduced macrophage TME infiltration and improved prognosis [142].

Despite the high levels of immune infiltrate, many cases of HPV^+^OPSCC continue to progress, indicating continuous immune evasion. OPSCC cells do this primarily by taking advantage of immune checkpoints, which are natural inbuilt mechanisms for the host immune system to ensure self-tolerance [165]. Two prominent examples are CTLA-4 (cytotoxic T-lymphocyte associated protein 4) and PD-1 (programmed cell-death protein 1). CTLA-4, which is commonly expressed on Tregs to downregulate T cell-mediated responses and is stimulated by tumour cells to cause T cell exhaustion. PD-1, on the other hand, is expressed on activated T and B lymphocytes to similarly limit T cell function. The PD-1 and its ligand (PD-L1) seem to play a substantial role in HPV^+^OPSCC development since higher levels of PD-L1 have been found in HPV^+^OPSCC [129]. Both the PD-1 and CTLA-4 systems have been successfully targeted with directed humanised antibodies and have been the cornerstone for revolutionised HNSCC treatment.

### 2.3. Immune Checkpoint Inhibitors

ICIs combat the elements of the TME which subvert the immune system’s efforts to restrain tumour proliferation [166]. The vast heterogeneity in cancer somatic mutations creates challenges in designing targeted therapies aimed at individual mutations; in contrast, ICIs have a broad scope as targeted cancer therapeutics [167]. Indeed, ICIs targeting CTLA-4 and PD-1 have shown clinical activity against HNSCC, advanced melanoma, renal cell carcinoma, non-small-cell lung cancer, Hodgkin’s lymphoma, endometrial cancer, and bladder cancer [167,168]. Recently, the approval of relatlimab (an ICI targeting LAG-3) based on a study showing that combined relatlimab with anti-PD-1 doubled the progression free survival of advanced melanoma patients compared to patients receiving PD-1 monotherapy highlights the growing therapeutic potential of ICIs [169].

The introduction of ICIs was a landmark turning point for targeted therapy in HNSCC. Several key clinical trials, including KEYNOTE-012 [170], KEYNOTE-040 [171], and CHECKMATE-141 [172], established the benefit of pembrolizumab and nivolumab in treating R/M HNSCC refractory to platinum base chemotherapy regardless of HPV status. However, subgroup analysis by HPV status also revealed an improved objective response rate (ORR) in HPV^+^ when compared to HPV^−^ tumours, such as in the Phase 1b KEYNOTE-012 trial when treated with pembrolizumab (25% to 14%) [170]. This was corroborated by KEYNOTE-012, which trialled fixed-dose pembrolizumab and demonstrated an ORR of 32% in HPV^+^ tumours and 14% in HPV^−^ tumours [173]. KEYNOTE-40, a Phase 3 clinical trial of Pembrolizumab, failed to identify a difference in ORR based on HPV status [171], while CHECKMATE-141 concluded that nivolumab improved OS in HPV^+^ tumours [172].

KEYNOTE-48, a Phase-3 clinical trial comparing pembrolizumab as a first line treatment in R/M HNSCC against standard of care EXTREME regime (platinum-based chemotherapy, 5-fluorouracil, and cetuximab), confirmed the superiority of pembrolizumab as initial treatment for this condition. However, HPV^+^ tumours were matched across treatment arms, attenuating the ability to determine the benefit of pembrolizumab by HPV status [174].

Neoadjuvant delivery of ICIs is also being trialled. CHECKMATE-358, a Phase 1/2 trial, concluded that HPV^+^HNSCC tumours demonstrated improved response to neoadjuvant nivolumab compared to HPV^−^HNSCC (23.5% vs. 5.9%) [102]. Meta-analysis of 12 clinical trials found that HPV^+^ tumours had an overall 1.29-fold higher likelihood of responding to ICI therapy than HPV^−^ tumours [175]. These results established a precedence for further research into the efficacy of ICIs for HPV^+^OPSCC treatment specifically, several of which are still ongoing (Table 1).

While there may be potential benefits of ICIs in the HPV^+^OPSCC cohort, these therapies unfortunately also carry the risk of immune-related adverse effects (irAEs). Common adverse effects observed include gastrointestinal (diarrhoea, nausea, vomiting), dermatological (dermatitis, rash), endocrinological (hypothyroidism), and systemic effects (fever, fatigue, headache, myalgia, arthralgia) [176]. The incidence of any irAEs in HNSCC patients on ICIs is approximately 57–67%, while more severe irAEs occur in 8–17% of HNSCC patients [177]. Severe irAEs may impact cardiopulmonary, hepatic, renal, neurological, gastrointestinal, and haematological systems. The mortality rate from irAEs is reported to be 0.3–1.03%, predominantly associated with gastrointestinal toxicity associated with Ipilimumab and pulmonary toxicity in PD-1 inhibition. These present further important considerations in establishing the most appropriate immunotherapy regimen for HPV^+^OPSCC.

Additionally, these drugs are associated with financial toxicity, representing a significant burden to the healthcare system. A systematic review determined that nivolumab was not a cost-effective therapy in the setting of R/M HNSCC over standard of care therapy (cetuximab, docetaxel, or methotrexate) given the high cost per quality-adjusted life years ($140,672 per QALY) [178]. While there are limitations to the estimations and calculation methods employed, it is important to keep in mind the financial burden of novel therapies as they are introduced into the clinical environment.

## 3. Emerging Therapeutic Strategies

Several new immune targets have been identified that present new opportunities to develop HPV-specific therapies.

### 3.1. Small Molecule Immunotherapy

#### 3.1.1. RTK

Receptor tyrosine kinases (RTKs) are a family of cell surface receptors which regulate a range of homeostatic cellular processes including intercellular communication, metabolic functions, and cell differentiation [179]. The majority of HNSCCs are found with overexpression of many RTKs, including HER, FGFR, and VEGFR families. EGFR (or HER1) has particularly been shown to be highly expressed in most HNSCCs, leading to the investigation and approval of cetuximab, an anti-EGFR monoclonal antibody, for locally-advanced and R/M HNSCC irrespective of HPV status [180,181]. The ORR to cetuximab as a single agent is limited (~13%) [182] but when used with PD-1 inhibitors, its efficacy improves to 45% in treating R/M HNSCC [183].

However, several studies have demonstrated cetuximab’s low efficacy in HPV^+^OPSCC (reviewed in [184]). Two major Phase 3 trials found cetuximab to be inferior to standard cisplatin treatment in combination with radiotherapy for HPV^+^OPSCC [185,186]. A recent meta-analysis further revealed that the combination of cetuximab and a PD-1 inhibitor improved the OS rate of HPV^−^OPSCC only compared to PD-1 inhibitor monotherapy, while patients with HPV^+^OPSCC did not experience the same benefit [187]. Another Phase 3 trial investigated adding panitumumab, another anti-EGFR monoclonal antibody, to chemotherapy and found improved OS only in HPV^−^OPSCC [188]. This may potentially be due to the reduced EGFR expression generally found in HPV^+^OPSCC [189,190,191]. However, a correlation between EGFR expression and therapeutic response has never been demonstrated in HNSCC [192,193]. Further research is required to fully elucidate the mechanism behind this poor response in HPV^+^OPSCC.

Interestingly, HPV^+^OPSCC has been found to express higher levels of HER2 and HER3 [194,195]. One study discovered that HPV^+^ cells relied on HER3 specifically for cellular proliferation and that HER3 expression was regulated by HPV E6 and E7 oncogenes, indicating that HER3 might be a viable therapeutic target [196]. HER3 signalling has also been shown to be important in HPV^−^OPSCC as upregulation contributes to cells acquiring cetuximab resistance [197,198].

An anti-HER3 monoclonal antibody, CDX-3379 (previously called KTN3379), initially demonstrated promising results in pre-clinical trials. In a Phase I window trial, tumour regression was noted in 42% of patients with newly diagnosed HNSCC, including two of the three HPV^+^ patients [199]. A Phase 1b trial found one patient with R/M HNSCC who had tumour progression on cetuximab to have a prolonged complete response when CDX-3379 was added to cetuximab [200]. However, in a Phase 2 clinical trial using CDX-3379 and cetuximab in patients with HPV^−^ R/M HNSCC, the limited overall response rate (6.7%) and significant adverse effects observed necessitated closure of the trial [201]. Further research may be able to identify predictive biomarkers for responses to anti-HER3 therapy and explore other agents such as an emerging anti-HER3 antibody-drug conjugate, U3-1402, which has shown anti-tumour activity in breast and lung cancer [202,203].

#### 3.1.2. STAT

The signal transducer and activator of transcription (STAT) proteins are a group of transcription factors with large influence on tumour proliferation and chemotherapy resistance [204]. These have been found to promote carcinogenesis by facilitating metabolic alterations which modulate gene expression and cytokine/growth factor signalling pathways. Additionally, they can reprogram immune cells within the TME to favour immunosuppression [205] and induce HNSCC resistance to standard therapies.

Both HPV^+^OPSCC and HPV^−^OPSCC have been found to have overactivated STAT protein signalling [206]. Hence, STAT inhibitors have long been a research focus for HNSCC regardless of HPV status. Several studies have demonstrated that the combination of STAT inhibitors with radiotherapy [207,208,209], chemotherapy [210], anti-CTLA-4 and anti-PD-L1 ICIs [211,212], and anti-EGFR therapies like cetuximab [213] can improve tumour response and ameliorate resistance. As a single agent, C188-9, a small-molecule STAT3 inhibitor, was found to have an in vitro anti-tumour effect on HNSCC cells [214].

In various HPV-related cancers, the JAK/STAT signalling pathway has also been found to be a primary pathway manipulated by HPV oncoproteins to facilitate ongoing viral replication [215]. Cervical cancer cells have been found to have high levels of STAT3 expression, and small molecule inhibition of STAT has been shown to facilitate cervical cancer cell death in vitro [216]. STAT inhibitors have not yet been trialled specifically in HPV^+^OPSCC but may prove to be an area of further interest.

#### 3.1.3. STING

Stimulator of interferon genes (STING) is an important protein which, when activated, phosphorylates interferon regulatory factor 3 (IRF3) and promotes interferon production [205]. This has been found to be crucial in mediating innate immunity anti-viral and anti-tumour responses [217]. Recent research has found HPV oncogenes E7 and E5 to strongly antagonise the STING pathway in order to facilitate immune evasion [80,117,118,119]. Therefore, the development of STING activators may theoretically improve tumour susceptibility to immune destruction. Indeed, use of a STING activator in several cancer types, including cervical cancer [218], has demonstrated promising results in reducing tumour growth [219,220]. While it has not been used as a single agent in HPV^+^OPSCC, evidence suggests that preclinical co-administration of STING activators may improve tumour responses to ICIs [221] or cetuximab [222] in HPV^+^OSPCC cells.

#### 3.1.4. PPAR

Peroxisome proliferator-activated receptors (PPARs) are gene transcription regulators belonging to the nuclear hormone receptor family. The three identified isoforms (PPARα, PPARβ/δ, and PPARγ) are uniquely expressed by distinct tissue types but all contribute to maintaining homeostatic metabolic activity [223]. They have also been implicated in the development of various diseases like atherosclerosis, Type 2 diabetes, and cancer. PPARs have also been identified also have a role in hepatocellular [224], pancreatic [225], lung [226], and breast carcinogenesis [227]. In cervical cancer, some evidence has found PPARγ to be anti-proliferative. Not only is it downregulated in cervical cancer cells [228], but treatment with a PPARγ agonist seems to enhance apoptosis [229] while PPARγ inhibition supports tumour progression [230]. One study posits that the mechanism for this involves HPV16 E7 inhibiting PPARγ expression via an increase in miR-27b to facilitate tumour proliferation [230].

Little research has gone into PPARs and HPV^+^OPSCC specifically. However, one paper found that fenofibrate demonstrated substantial anti-tumorigenic effects on HPV^+^ HNSCC cells, especially in combination with cisplatin administration [231]. Fenofibrate is an established drug for dyslipidaemia known to act on PPARα pathways; however, it is unclear if PPARα is similarly involved in its anti-tumour activity. The same study found increased p53 activation and immune cell infiltration with fenofibrate [231], which makes it an interesting research target for future HPV^+^OPSCC therapy.

#### 3.1.5. AHR

Aryl hydrocarbon receptor (AHR) is a ligand-activated transcription factor that, when chronically activated, has a strong role in supporting tumour invasion, migration, and metastasis including in HNSCC [232,233,234,235]. Additionally, AHR can have immunosuppressive effects on CD8+ TILs by inducing PD-1 and antigen-presenting cells [236]. No research has been published on AHRs in HPV^+^OPSCC; however, when a natural AHR ligand, indole-3-carbinol, was administered to cervical cancer cells, it was found to hinder cell proliferation [237], indicating that this may be a target for future exploration.

#### 3.1.6. NET Based Therapies

##### Polyanions

Potential polyanion agents aim to capitalise on electrostatic interaction between highly cationic components of NETs (i.e., histones) to inhibit pro-tumorigenic effects, namely tumour cell camouflage, migration, and dormant tumour cell reactivation. They may also interfere with pro-tumorigenic histone-dependent pathways, including TLR4/histone-dependent TME immunosuppression, histone-dependent endothelial, and platelet activation and thrombosis, all of which confer tumour cell survival and metastatic ability. Heparin particularly has been shown to degrade NETs. These polyanionic compounds include STC3141 (methyl β-cellobioside per-O-sulfate), unfractionated heparin, and enoxaparin [136,238,239].

#### 3.1.7. NET Modulators or Preventors

Several agents have been shown to inhibit NET expression or production, including sivelestat, a neutrophil elastase inhibitor. NETosis and NET formation is dependent on neutrophil elastase and the pro-tumorigenic role of neutrophil elastase has been identified in several cancers [240,241,242]. In a murine model of colorectal cancer, sivelestat has been shown to suppress liver metastasis.

The COX-1 inhibitor, aspirin, has been shown to reduce neutrophil tissue invasion and NET production, believed to be caused by inhibition of CCL5 (RANTES) and CXCL4 (PF4) release, both shown to increase neutrophil chemotaxis. Several studies have demonstrated the anti-metastatic benefit produced through attenuation of NET production by COX-1 inhibition [243,244,245].

Studies have demonstrated that metformin decreases NET production, even in the presence of NET stimulants and recent evidence in hepatocellular carcinoma and pancreatic cancer confirmed that metformin reduced the production of NETs and the metastatic potential of these two cancers [246,247,248]. This has subsequently been corroborated in preclinical animal models [249].

#### 3.1.8. NET Degraders

Dornase Alfa (rhDNase 1) is a recombinant human deoxyribonuclease that can selectively cleave DNA. Park and colleagues have demonstrated that ability of rhDNase 1 to reduce the 4T1 breast cancer metastatic burden in a preclinical model, while further preclinical studies have shown that it can reduce tumour progression in pancreatic cancer [157,247].

## 4. Conclusions

HPV^+^ and HPV^−^OPSCC are very different tumours and the immune system plays an integral role in this difference. Personalised treatment strategies remain to be determined; however, greater insight into the specific interaction between the HPV virus and the immune system for this cancer will help define immunotherapeutic strategy. As our understanding of this relationship improves, there exists an exciting potential for the development of novel small molecule immunotherapies that may help to de-escalate standard of care interventions, improving survival and attenuating functional deficits associated with treatment.

## Figures and Tables

**Table 1:** Ongoing clinical trials related to HPV^+^OPSCC.

Phase	Trial	Population	Therapy	Objectives	Status
2	NCT03799445	Advanced HPV^+^ HNSCC	Concurrent ipilimumab + nivolumab + RT	Complete response rate Progression free survival Toxicity	Recruiting
2	NCT03383094	Intermediate/high-risk HPV^+^ locoregionally-advanced HNSCC	Concurrent and adjuvant pembrolizumab + RT vs. RT + cisplatin	Progression-free survival Overall survival Toxicity	Recruiting
2	NCT04988074	Advanced HPV^+^OPSCC	Neoadjuvant cemiplimab + TORS/RT +/− chemotherapy	Progression-free survival Quality of life Swallow function Overall survival	Recruiting
2	NCT04867330	HPV^+^OPSCC	Toripalimab + docetaxel/cisplatin	Progression-free survival	Recruiting
3	NCT04116047	Intermediate/high-risk HPV^+^OPSCC	Durvalumab vs. chemoradiotherapy	Overall survival Event-free survival Toxicity Quality of life	Recruiting
2	NCT03410615	Intermediate-risk HPV^+^ locoregionally-advanced OPSCC	Durvalumab + RT + adjuvant Durvalumab vs. Durvalumab + RT + adjuvant Tremelimumab and Durvalumab vs. Cisplatin + RT	Event-free survival Functional assessment Locoregional failure Overall survival	Active, not recruiting
2	NCT03829722	High-risk HPV^+^OPSCC	Concurrent nivolumab + RT + carboplatin	Progression-free survival Overall survival Toxicity	Active, not recruiting
2	NCT03107182	Locoregionally-advanced HPV^+^OPSCC	Nivolumab/Nab-paclitaxel/Carboplatin Induction Chemotherapy followed by Response-stratified Locoregional Therapy	Deep response rate Adverse events Progression-free survival Overall survival	Active, not recruiting
2	NCT03838263	High-risk HPV^+^OPSCC	Neoadjuvant nivolumab + chemoradiotherapy vs. chemoradiotherapy alone	Feasibility assessment Adverse events Objective response rate Overall survival Progression-free survival	Active, not recruiting
2/3	NCT03952585	Early-stage HPV^+^OPSCC	Concurrent reduced-dose RT + either nivolumab or cisplatin	Progression-free survival Quality of life Overall survival	Suspended
3	NCT03811015	Intermediate risk locally-advanced HPV^+^OPSCC	Definitive chemoradiotherapy followed by maintenance nivolumab	Overall survival Progression-free survival	Recruiting

RT = radiotherapy. TORS = transoral robotic surgery

## Data Availability

Data for this review article was sourced from reporting manuscripts.

## Abbreviations

HPV^+^OPSCC	Human Papillomavirus Positive Oropharyngeal Squamous Cell Carcinoma
HPV^−^OPSCC	Human Papillomavirus Negative Oropharyngeal Squamous Cell Carcinoma
